# Tetrahydrocannabinol and cannabidiol medicines for chronic pain and mental health conditions

**DOI:** 10.1007/s10787-022-01020-z

**Published:** 2022-07-07

**Authors:** Jeremy D. Henson, Luis Vitetta, Sean Hall

**Affiliations:** 1 Prince of Wales Clinical School, University of NSW, Sydney, NSW 2052 Australia; 2 Medlab Clinical Ltd, Sydney, NSW 2015 Australia; 3 Faculty of Medicine and Health, The University of Sydney, Sydney, NSW 2006 Australia

**Keywords:** Tetrahydrocannabinol, Cannabidiol, Pain, Stress, Anxiety, Depression, Insomnia

## Abstract

Combination tetrahydrocannabinol (THC)/cannabidiol (CBD) medicines or CBD-only medicines are prospective treatments for chronic pain, stress, anxiety, depression, and insomnia. THC and CBD increase signaling from cannabinoid receptors, which reduces synaptic transmission in parts of the central and peripheral nervous systems and reduces the secretion of inflammatory factors from immune and glial cells. The overall effect of adding CBD to THC medicines is to enhance the analgesic effect but counteract some of the adverse effects. There is substantial evidence for the effectiveness of THC/CBD combination medicines for chronic pain, especially neuropathic and nociplastic pain or pain with an inflammatory component. For CBD-only medication, there is substantial evidence for stress, moderate evidence for anxiety and insomnia, and minimal evidence for depression and pain. THC/CBD combination medicines have a good tolerability and safety profile relative to opioid analgesics and have negligible dependence and abuse potential; however, should be avoided in patients predisposed to depression, psychosis and suicide as these conditions appear to be exacerbated. Non-serious adverse events are usually dose-proportional, subject to tachyphylaxis and are rarely dose limiting when patients are commenced on a low dose with gradual up-titration. THC and CBD inhibit several Phase I and II metabolism enzymes, which increases the exposure to a wide range of drugs and appropriate care needs to be taken. Low-dose CBD that appears effective for chronic pain and mental health has good tolerability and safety, with few adverse effects and is appropriate as an initial treatment.

## Introduction

Tetrahydrocannabinol (THC) and cannabidiol (CBD) combination medicines and CBD-only medicines are prospective new treatments for chronic pain, stress, anxiety, depression, and insomnia, which are all medical conditions in need of better therapeutics. Both THC/CBD combination and CBD-only medicines could provide effective new treatment options for pain and mental health, respectively, and both have good safety and tolerability profiles relative to the current treatments. This review assesses the potential of THC/CBD combination medicines and CBD-only medicines for treating chronic pain, stress, anxiety, depression, and insomnia based on the clinical evidence for their efficacy, safety, and tolerability. We also review the pharmacokinetics and mechanisms of action of THC and CBD, an understanding of which is necessary for their optimal use.

## Current use of tetrahydrocannabinol and cannabidiol medicines

There are currently four cannabis-based pharmaceuticals (apart from the cannabis plant) that are approved by a major regulatory body, such as the Food and Drug Administration (United States) or the Medicines and Healthcare products Regulatory Agency (United Kingdom) (Advisory Council on the Misuse of Drugs (ACMD). 2020; Schlag et al. 2020; Borgelt et al. 2013):

i. Dronabinol, synthetic delta-9-tetrahydrocannabinol (THC) administered as a per oral capsule for the treatment of anorexia and weight loss in patients with acquired immune deficiency syndrome; and chemotherapy-induced nausea and vomiting.
ii. Nabilone, a synthetic analog of delta-9-tetrahydrocannabinol administered as a per oral capsule for the control of chemotherapy-induced nausea and vomiting.
iii. Epidyolex, a per oral administered solution of botanically extracted cannabidiol (CBD) that is used as an adjunct to clonazepam for the treatment of seizures associated with Lennox-Gastaut syndrome or Dravet syndrome.
iv. Nabiximols, an oral-buccal spray administered combination of botanically extracted THC and CBD in an ethanol and polyethylene glycol vehicle for the treatment of spasticity and/or neuropathic pain in adults with multiple sclerosis, and for the treatment of intractable cancer pain.

Unsanctioned use of THC and/or CBD medicines appears to be high, indicating a demand not met by that, which can be currently provided with expert guidance and good manufacturing practice products. For example, in the United Kingdom (UK), over one-third of patients with at least some conditions have used non-prescribed cannabis-based preparations and over three-quarters of UK adults would take cannabis-based medicines if prescribed by their doctor (Statista 2020). In western countries, although over 10% of adults have tried cannabidiol, less than 1% of this is prescribed by a medical practitioner and under 5% is obtained from a pharmacy, with most being purchased from online suppliers (Moltke and Hindocha 2021).

## Mechanisms of action of tetrahydrocannabinol and cannabidiol

### Tetrahydrocannabinol (THC)

Delta-9-tetrahydrocannabinol (THC) is the primary psychoactive compound of cannabis and exerts its psychoactive and analgesic effects mainly by its partial agonist activity at the endogenous cannabinoid (endocannabinoid) receptors, cannabinoid receptors type 1 (CB_1_) and type 2 (CB_2_) (Pertwee 2008a; Lucas et al. 2018). Because THC is only a partial CB_1/2_ agonist it may sometimes act as a competitive inhibitor of endocannabinoids, resulting in decreased CB_1/2_ signaling, which may be the cause of the anxiogenic effects of THC (Hillard et al. 2016). The two main endocannabinoids, N-arachidonylethanolamine (anandamide; AEA) and 2-arachidonoylglycerol (2-AG) are synthesized as required by cleavage of membrane phospholipids, which is usually induced by depolarization (neurons) and/or Ca^+2^ influx. In the nervous system, synthesis occurs in the post-synaptic terminals and AEA and 2-AG feedback in a retrograde manner to CB_1/2_ on presynaptic membranes, inhibiting further neurotransmitter release (Blessing et al. 2015; Morena et al. 2016).

CB_1_ are G-protein coupled receptors and are found mainly on presynaptic terminals of excitatory glutamatergic neurons and inhibitory gamma-aminobutyric acid (GABAergic) interneurons, as well as serotonergic, noradrenergic, and dopaminergic neurons (Morena et al. 2016). CB_1_ are highly expressed in the cerebral cortex, hippocampus, amygdala, basal ganglia, hypothalamus, septum, cerebellum, parts of the brainstem, the dorsal horn of spinal cord, sympathetic nervous system, enteric nervous system and nociceptors (Devesa and Ferrer-Montiel 2014; Mackie 2005; Zou and Kumar 2018), which includes parts of the nervous system involved in stress, anxiety, mood, memory, learning, cognition, reward, addiction, appetite, sleep and pain (Zou and Kumar 2018). In animal models, CB_1_ signaling both in the peripheral terminals of nociceptors and in spinal neurons play a substantial role in cannabinoid induced analgesia and reduction of pain sensitization (Agarwal et al. 2007; Furuse et al. 2009; Wang et al. 2014; Yang et al. 2016).

The highest expression of CB_2_ is in immune cells, including microglia, where they modulate cell migration and cytokine release. CB_2_ signaling can inhibit pain signaling and sensitization by inducing the secretion of mediators that alter primary afferent neuron responsiveness to noxious stimuli and by inhibiting the secretion of proinflammatory cytokines (Sun et al. 2019). CB_2_ are also expressed by neurons in the limbic areas of the brain and inhibit GABA, dopamine, and glutamate neurotransmitter release (García-Gutiérrez et al. 2012; Manzanares et al. 2018; Zoppi et al. 2014).

### Cannabidiol (CBD)

The main non-psychomimetic and non-addictive active compound of cannabis, CBD, may be useful for treating numerous neuropsychiatric disorders (De Gregorio et al. 2019), including chronic pain (Costa et al. 2007). CBD increases CB_1_ and CB_2_ signaling by increasing AEA levels, which it does (in humans) by competitively inhibiting AEA binding to fatty acid-binding proteins that transport hydrophobic AEA across the aqueous space from the post-synaptic membrane to Fatty-Acid Amide Hydrolase (FAAH) in the post-synaptic endoplasmic reticulum (Elmes et al. 2015). Clinically, CBD treatment increases serum AEA levels in schizophrenia patients (Leweke et al. 2012). In vitro, CBD is a negative allosteric modulator of CB_1_ (Laprairie et al. 2015; McPartland et al. 2015) and CB_2_ (Martínez-Pinilla et al. 2017), thereby non-competitively antagonizing THC and endogenous CB_1/2_ agonists. Clinically, CBD does antagonize some actions of THC. However, CBD has different clinical effects to recombinant and other antagonists of CB_1/2_ (McPartland et al. 2015), and the overall clinical effect of CBD is to increase CB_1/2_ signaling (Blessing et al. 2015; Campos et al. 2013; Fogaça et al. 2018). CBD is also an antagonist of GPR55, which is an endocannabinoid receptor with multiple putative clinical actions (de Almeida and Devi 2020).

CBD also acts on non-endocannabinoid receptors. CBD acts as a potent agonist at serotoninergic 5-HT_1A_ receptors, which contributes to the effectiveness of low-dose CBD for reducing stress, anxiety, and pain (Campos and Guimarães 2008; de Almeida and Devi 2020; Lee et al. 2017; Viudez-Martínez et al. 2018). The anxiolytic and anti-depressant effects of CBD may also be in part due to the partial agonism of CBD at Dopamine D2 receptors (de Almeida and Devi 2020). By stimulating peroxisome proliferator-activated receptor gamma (PPARγ), CBD inhibits inflammation, neuroinflammation and excitotoxicity, which may prevent the development of mental health disorders (García-Bueno et al. 2007). CBD binds and desensitizes the transient receptor potential vanilloid member 1 (TRPV1) (Costa et al. 2004), a mediator of pain signaling. The analgesic effect of CBD may also result from CBD having allosteric modulator activity at Mu and Delta opioid receptors (de Almeida and Devi 2020). CBD is an agonist at adenosine A_1_ and A_2A_ receptors, which may contribute to the somnogenic and anti-inflammatory effects of CBD (de Almeida and Devi 2020). CBD also inhibits sodium and calcium channels, which would dampen nerve excitability and may contribute to decreasing pain hypersensitivity and seizures (de Almeida and Devi 2020).

## Efficacy of THC/CBD and CBD-only medicines for pain and mental health conditions

### Chronic pain

After over 2000 years of medicinal use by many cultures around the world, the most common indication for cannabis and cannabis-based medicine (CBM) use is still to relieve chronic pain (Boehnke et al. 2019; Ergisi et al. 2022). The National Academy for Science, Engineering and Medicine in the United States of America reviewed the clinical evidence in 2017 and found that there was substantial evidence that CBM was effective for chronic pain treatment (National Academies of Sciences, Engineering, and Medicine. 2017). However, the UK National Institute for Health and Care Excellence (NICE) review of the literature in 2019 found that CBM was only 17% better than placebo (54% compared to 46%) at providing a 30% reduction in chronic pain (National Institute For Health And Care Excellence (NICE) Guidelines Updates Team. 2019). Part of the reason for these underwhelming results could be that response to CBM depends on the pain mechanism involved. A large observational study of 800 participants by the German pain registry found that Nabiximols (THC/CBD combination oral spray) provided no reduction in pain severity for nociceptive pain and a greater than 30% reduction in pain severity for neuropathic pain (Ueberall et al. 2019). This study used the painDETECT questionnaire (Freynhagen et al. 2006) to identify the presence of the neuropathic pain mechanism; however, the painDETECT questionnaire was devised before recognition of the nociplastic pain mechanism and does not distinguish between neuropathic and nociplastic pain. There are three pain mechanisms:

1. Nociceptive pain: actual or threatened tissue damage causing the activation of peripheral nociceptors.
2. Neuropathic pain: disease or lesion of the somatosensory system causing the pain.
3. Nociplastic pain: arising from altered nociception despite no clear evidence of nociceptive or neuropathic pain. This mechanism is commonly seen in fibromyalgia, chronic migraines and headaches, chronic primary visceral pain (such as irritable bowel syndrome), chronic primary musculoskeletal pain (such as chronic lower back pain), and complex regional pain syndrome (Fitzcharles et al. 2021; Kosek et al. 2021).

Considering the current evidence, a panel of 20 experts from nine countries have recommended the use of CBM for the neuropathic and nociplastic pain mechanisms and not nociceptive pain (Bhaskar et al. 2021). They also suggested that CBM could be considered if there was a substantial inflammatory component and that CBD-only medicine could be tried initially due to its better safety profile; however, all clinical evidence is for CBM containing THC and there is no randomized controlled clinical trial evidence for the use of CBD-only medicine for chronic pain (National Institute For Health And Care Excellence (NICE) guidelines updates team 2019; Bhaskar et al. 2021). Although, chronic pain is a common reason for self-prescribed CBD-only products (Moltke and Hindocha 2021) and the addition of CBD to THC medicines improves analgesic efficacy (Johnson et al. 2010).

### Chronic stress

Chronic stress causes considerable morbidity and economic loss that could be prevented by a safe, rapid and effective treatment (Henson et al. 2021). The stress response involves secretion of cortisol and catecholamines from the hypothalamus–pituitary–adrenal (HPA) axis and sympathetic nervous system, and prolonged activity is a substantial causal contributor to many diseases, including nociplastic pain conditions and mental health disorders (Eller-Smith et al. 2018; Henson et al. 2021; Sluka and Clauw 2016). The endocannabinoid system is a good therapeutic target for constraining the magnitude and duration of the stress response and therefore preventing stress-associated morbidities. Endocannabinoid signaling reduces the size of the stress response, returns cortisol and the HPA axis to basal levels, and enables habituation of the stress response to on-going or repeated stress. Independent of its action on the HPA axis, endocannabinoid signaling directly inhibits downstream effects of the stress response, such as fear, anxiety, depressive behaviors, hyperalgesia and inflammation, and restores stress-inhibited behaviors such as sleep and feeding (Henson et al. 2021).

CBD enhances endocannabinoid signaling and has several other activities that also constrain the stress response (Sect. 3.2). Clinically, CBD has proven to be a safe and effective treatment for stress, including in seven double-blind placebo controlled clinical trials (Appiah-Kusi et al. 2020; Crippa et al. 2004; Das et al. 2013; Karniol et al. 1974; Zuardi et al. 1993, 2017, 1982) on 232 participants, and one partly controlled clinical trial on 120 participants (Crippa et al. 2021). All these studies found CBD to be effective in reducing the stress response and its manifestations of fear, anxiety, depressive behaviors, or burnout. Two of these studies (Crippa et al. 2021; Zuardi et al. 1993, 2017) included a comparator (benzodiazepine and/or 5HT_1A_ agonist) arm, and both found CBD to be non-inferior to the effectiveness of the pharmaceutical drug. These clinical trial findings are well supported by extensive preclinical evidence (Henson et al. 2021), and also the prevalent unregulated medicinal use of CBD for treating stress. Approximately, 5% of the adult population have used CBD to treat stress, and 90% have found it to be effective (Moltke and Hindocha 2021).

### Anxiety disorders

Anxiety disorders generally involve an excessive anticipation of future threats that is accompanied by excessive fear (American Psychiatric Association 2013), and are the most prevalent psychiatric disorders, with a 12-month prevalence of 18% (Kessler et al. 2005). Better therapeutic agents for anxiety are needed (Wright et al. 2020) because only 50% of patients respond to the current medications (Davidson et al. 2004; Liebowitz et al. 2005; Stein et al. 1998; Van Ameringen et al. 2001), many of these responders are still clinically symptomatic, and the side effects of these medications often interfere with patient functioning.

Preclinical studies support the efficacy of low dose CBD for anxiety. Several animal models and behavioral tests have shown that low dose CBD delivers anxiolytic effects similar to diazepam but is anxiogenic at high doses (Guimarães et al. 1990; Onaivi et al. 1990; Papagianni and Stevenson 2019; Wright et al. 2020). The first clinical evidence that CBD was anxiolytic were studies demonstrating that CBD attenuated the anxiogenic effects of THC in healthy volunteers (Karniol et al. 1974; Zuardi et al. 1982). There have been three clinical trials of CBD for treating anxiety disorders. One placebo controlled study used the Simulation Public Speaking Test to induce anxiety in patients with social anxiety disorder (SAD) and found that a single dose of CBD (400–600 mg) significantly decreased symptoms of performance discomfort, anxiety and cognitive impairment (Bergamaschi et al. 2011; Crippa et al. 2011). Masataka et al. showed that relative to placebo, four weeks treatment with CBD, 300 mg/day, significantly reduced anxiety in 18–19 year old participants with SAD and avoidant personality disorder (Masataka 2019). A retrospective clinical trial that included any type of anxiety disorder, found that a lower dose of CBD (25 mg/day) was also effective (Shannon et al. 2019).

### Depression

There are no randomized controlled trials of CBD for depression however three open-label clinical trials on a total of 434 participants each found that CBD significantly decreased depressive symptoms (Beale et al. 2018; Gulbransen et al. 2020; Solowij et al. 2018). Two observational studies of a total of 2649 participants both found that depression was a common indication for self-prescribed CBD (Corroon and Phillips 2018; Moltke and Hindocha 2021). Preclinical models have demonstrated CBD to have anti-depressant effects that result from increased CB_1_ and 5-HT_1A_ signaling (Bonaccorso et al. 2019).

### Chronic insomnia

Chronic insomnia is one of the most prevalent complaints in primary care and is without an appropriate pharmacological treatment (Morin and Benca 2012). Early work on whole plant cannabis showed that cannabis may help to improve sleep onset latency and sleep maintenance insomnia (Babson et al. 2017). Although insomnia is a common reason for self-prescribed CBD (Moltke and Hindocha 2021), there has been only a few randomized controlled trials (RCT) of CBD for insomnia. An early double-blind RCT found that 160 mg CBD in participants suffering from insomnia improved their duration of sleep significantly better than placebo and was non-inferior to 5 mg nitrazepam (Carlini and Cunha 1981). A recent retrospective case series of 25 psychiatric clinic patients with sleep complaints showed only a mild improvement in the Pittsburgh Sleep Quality index with 25 mg of CBD after dinner (Shannon et al. 2019); however, because the usual time to peak plasma levels of CBD after oral administration can be 4–5 h (Taylor et al. 2018) the dosing in this study may not have been early enough to improve sleep onset latency. CBD (300 mg) does not acutely affect the sleep cycle in healthy people without insomnia (Linares et al. 2018).

THC is generally not appropriate for chronic insomnia due to habituation of any short-term benefit it provides, reports of THC worsening sleep onset latency and daytime sleepiness (Babson et al. 2017; Gorelick et al. 2013; Vaughn et al. 2010) and an inappropriate safety and tolerability profile for this indication (Sect. 5). However, THC may be potentially useful for other sleep disorders such as obstructive sleep apnea (Babson et al. 2017).

## Safety, tolerability, and pharmacokinetics

### THC/CBD combination medicine safety and tolerability

Two literature reviews of a combined total of 55 clinical trials of the equimolar THC/CBD oro-buccal spray, Nabiximols (Prieto González and Vila Silván 2021; Torres-Moreno et al. 2018), found that the adverse events of Nabiximols led to an approximately twofold higher discontinuation rate than placebo. Common adverse events that were significantly associated with Nabiximols consisted of:

1. Dizziness and/or vertigo
2. Dry mouth
3. Fatigue
4. Nausea and/or vomiting
5. Somnolence
6. Impaired balance and/or ataxia
7. Memory impairment
8. Feeling intoxicated (Prieto González and Vila Silván 2021; Torres-Moreno et al. 2018).

These common adverse reactions were usually dose-proportional and demonstrated tachyphylaxis or tolerance and were minimized by commencing on a low dose of Nabiximols with gradual up-titration (Prieto González and Vila Silván 2021; Robson 2011; Torres-Moreno et al. 2018). Similar adverse event profiles were demonstrated for MC-1019 (Vitetta L. et al. unpublished) and LGP Classic 10:10 (Abelev et al. 2022), which have similar active pharmaceutical ingredient compositions to Nabiximols. For patients taking THC/CBD combination medicinal products, caution is required with the undertaking of potentially dangerous tasks, such as driving or the use of heavy equipment, because of the reported adverse reactions with Nabiximols of vertigo and ataxia combined with the known impairment of reaction times and motor coordination and increased risk of motor vehicle accidents that occurs with recreational cannabis (Asbridge et al. 2012; Fitzcharles et al. 2021, Robson 2011).

Serious adverse events are listed below; however, they were not significantly higher than in the placebo group and therefore may not be related to Nabiximols treatment (Prieto González and Vila Silván 2021; Torres-Moreno et al. 2018):

1. Nervous system events: seizures, balance disorder, paraesthesia, dizziness, tremor, and somnolence.
2. Psychiatric events: confusion/disorientation, depression, suicidal ideation, drug dependence, aggression, delusions, insomnia, agitation, irritability, paranoia, amnesia, hallucinations.
3. Musculoskeletal system events: muscle spasms.
4. Gastrointestinal system events: nausea and/or vomiting, diarrhea, liver function test abnormality/hepatocyte injury, and constipation.
5. Renal and urinary system events: urinary tract infection.
6. Respiratory system events: aspirational pneumonia.
7. Cardiac system events: ventricular bigeminy and syncope.
8. Blood and vascular system events: pancytopenia (cervical cancer patient), pulmonary embolism (bone metastasis patient) (Prieto González and Vila Silván 2021; Torres-Moreno et al. 2018).

Psychiatric adverse events were the most frequently reported serious adverse events. Of the psychiatric adverse events, 80% occurred in the first month of treatment and 96% resolved during the clinical trial (Robson 2011). Although the risk of psychiatric adverse reactions is not clear, prescribing THC/CBD combinations should be avoided in patients predisposed to depression, psychosis and suicide, and to patients under the age of 26 years, who (due to continuing brain development) may be at increased risk of psychiatric adverse reactions (Fitzcharles et al. 2021). There was no evidence that cognitive dysfunction occurred with long-term Nabiximols treatment (Prieto González and Vila Silván, 2021; Torres-Moreno et al. 2018). However, care still needs to be taken, given that cognitive impairment has been reported with long-term inhaled or ingested cannabis use (Honarmand et al. 2011).

Abuse or dependence with medicinal equimolar THC/CBD combinations is rare. This contrasts with recreational cannabis use where the dependence rate is 9% (which may be an overestimate due to the previous positive legal implications of dependence in the United States) (Prieto González and Vila Silván, 2021, Robson, 2011; Russo 2019; Torres-Moreno et al. 2018). Although THC, the main component of cannabis responsible for euphoria and dependence, comprises 50% of the cannabinoids in equimolar THC and CBD oro-buccal sprays, dependence may be negligible with medicinal use of these products due to a higher level of CBD that antagonizes the psychotropic and anxiogenic activity of THC, lower peak plasma THC levels compared to smoking or vaping, steady state dosing, and users whose motivation is symptom relief without euphoria or cognitive changes (Robson 2011). In clinical trials of Nabiximols, neither tolerance nor formal withdrawal syndrome occurred (Notcutt et al. 2012; Robson 2011; Wade et al. 2006). Sudden withdrawal from long-term use only caused mild and transient symptoms, such as disturbance of sleep, mood, or appetite (Wade et al. 2006). In clinical trials of Nabiximols, representing over 1500 patient-years of treatment experience, intoxication scores were low, euphoria only occurred in 2.2% of patients, there was one case of possible psychological dependence and no cases of abuse or diversion (Robson 2011). In a formal abuse liability study (Schoedel et al. 2011), a single dose of Nabiximols (4 sprays; 10.8 mg THC/10 mg CBD) did not differ from placebo. Higher single doses than normally prescribed did show abuse potential; however, the abuse potential was lower than equivalent doses of synthetic THC (dronabinol) (Schoedel et al. 2011) that was demonstrated to have very low abuse potential after 13 years of availability as a prescription drug (Calhoun et al. 1998; Mücke et al. 2018).

There were no reports of acute myocardial infarct or cerebral vascular accident (Prieto González and Vila Silván 2021; Torres-Moreno et al. 2018); however, given the potential for increased myocardial oxygen demand and heart rate and blood pressure changes, caution should be taken with prescribing THC/CBD combinations to patients with cardiovascular disease (Desai et al. 2017; Hackam 2015; Singh et al. 2018; Thomas et al. 2014; Volkow et al. 2014).

THC and/or CBD containing medicines should be avoided in pregnancy, lactation and couples who may be conceiving a child. Cannabinoids can cross the placenta and be transferred into breast milk. THC and CBD may also inhibit secretion of gonadotrophin-releasing hormone and impair reproductive processes such as the maturation of sperm and ovarian follicles, and ovulation (Fonseca and Rebelo 2021; Mourh and Rowe 2017; Vardaris et al. 1976).

There are substantial pharmokinetic and pharmacodynamic interactions between both THC and CBD and other drugs. THC and CBD are metabolized by CYP3A and CYP2C isozymes, and inducers and inhibitors of these enzymes will decrease and increase their bioavailability, respectively (Brown 2020; Brown and Winterstein 2019; Huestis 2007; Lucas et al. 2018). Polymorphisms that reduce CYP2C9 activity will also increase THC and CBD exposure (Brown 2020). THC and/or CBD inhibit several CYP450 enzymes, carboxylesterase I, Phase II metabolism enzymes and drug transporters such as P-glycoprotein, which increases the exposure to a wide range of drugs and appropriate care needs to be taken when changing the THC/CBD dose (Brown 2020; Brown and Winterstein 2019; Huestis 2007; Lucas et al. 2018). THC and CBD also inhibit estrogen synthesis and estrogen receptor signaling, which can make estrogen-based medicines and the oral contraceptive pill ineffective (Amaral et al. 2021). THC and/or CBD can impair, motor skills, and cause sedation; and care needs to be taken with medications with similar effects, such as opioids and benzodiazepines (Brown 2020; Lucas et al. 2018). THC and CBD may also impair the immune response. THC has been shown to reduce T-helper response and pro-inflammatory cytokines causing a clinically significant reduction in the immune response in mice (Brown 2020; Lucas et al. 2018). In humans, there is potential for a reduction in the immune response and a reduction in the effectiveness of anti-cancer immunotherapies (Bar-Sela et al. 2020; Taha et al. 2019).

### CBD-only safety and tolerability

Low-dose CBD (less than 150 mg/day) that appears effective for chronic pain and mental health conditions has a good tolerability and safety, with few adverse effects (Di Bartolomeo et al. 2021; Iffland and Grotenhermen 2017; Stark et al. 2021). In contrast to THC, CBD is not psychotomimetic and does not cause euphoria, intoxication, addiction, or cognitive or psychomotor impairment (De Gregorio et al. 2019; Pertwee 2008b; Zuardi 2008). Importantly, low-dose CBD has not been found to cause hepatocellular injury, which can be caused by doses of CBD over 600 mg/day (Naftali et al. 2017; Notcutt et al. 2004; Shannon et al. 2019; Shannon and Opila-Lehman 2016). Results from 49 clinical trials of CBD with oral dose of 10 mg to 1.5 g per day, showed CBD to be well tolerated with a good safety profile (Bergamaschi et al. 2011; Iffland and Grotenhermen 2017). Clinical trials have also confirmed that CBD has no potential for abuse or dependence (World Health Organization (WHO), Geneva, Switzerland: WHO. 2018; Babalonis et al. 2017; Hindocha et al. 2015; Schoedel et al. 2018). Caution needs to be taken regarding drug-drug interactions with low-dose CBD, especially with medicines metabolized by the cytochrome P450 pathways (see Sect. 5.1); although, most drug-drug interactions reported for CBD-only medications (so far) have been for higher doses (Brown and Winterstein 2019). Mild drowsiness and fatigue are common adverse effects of low-dose CBD (Brown and Winterstein 2019).

## Pharmacokinetics of THC and CBD

Common administration methods for THC and/or CBD include oral (ingestion), oro-buccal mucous membrane absorption or inhalation (Britch et al. 2021; Millar et al. 2018; Silmore et al. 2021; Taylor et al. 2018; Vitetta et al. 2021; Moltke and Hindocha 2021). Because THC and CBD have poor aqueous solubility, ingestion delivers poor and erratic absorption, and most of the THC and CBD that is absorbed is modified by first-pass metabolism, which reduces systemic bioavailability to only 6% (Fasinu et al. 2016; Millar et al. 2018; Lucas et al. 2018). For CBD, systemic exposure can be improved fourfold by ingestion with a high-fat meal (Taylor et al. 2018) and is fivefold higher in patients with severe hepatic impairment (Taylor et al. 2019); with more modest increases in systemic exposure and time-to-peak-plasma-concentration for THC (Lunn et al. 2019). Gastrointestinal absorption is improved with a high-fat meal because the fatty acids mix with bile salts in the small intestine creating micelles that carry THC and CBD into intestinal epithelial cells and the portal circulation (Mozaffari et al. 2021). The portal circulation can be bypassed by oro-buccal absorption of THC and CBD; however, attempts to do this with sublingual THC and/or CBD has not reduced first-pass metabolite levels, which suggests poor mucous membrane absorption (Guy and Flint 2004). Oro-buccal absorption may be enhanced by biomimicry of the natural intestinal micelles, using synthetic nano-micellular preparations of THC and CBD (Vitetta et al. 2021). Inhalation is a faster method of administration, smoking or vaping THC/CBD has a time-to-peak-plasma-concentration (*T*_max_) ≤ 5 min, and bioavailability is around 30%, which is 25% higher than ingestion with a high fat meal (Ohlsson et al. 1986). The more rapid absorption of inhalation, causes a higher peak plasma THC level and increased adverse effects (Guy and Flint 2004). These increased adverse effects can be dose limiting, and together with the toxic oxidation products produced by the high temperatures involved, make inhalation unsuitable (Russo 2019).

The single-dose half-life of THC and CBD is 2—3 h (Lunn et al. 2019; Millar et al. 2018; Stott et al. 2013); however, THC and CBD accumulate in tissues, especially adipose tissues, due to their lipophilicity, and after multiple doses, their half-life is 2–5 days (Millar et al. 2018; Huestis 2007). THC and CBD bind to blood cells and proteins and have a high apparent volume of distribution of 6.4 L/kg and 32 L/kg, respectively (Fasinu et al. 2016; Ohlsson et al. 1986). Administration of oral, sublingual and oro-buccal THC and/or CBD at therapeutic doses for chronic pain, stress, anxiety, and insomnia provide plasma THC and CBD levels in the order of 1–10 ng/ml for THC, and 1–30 ng/mL for CBD (Britch et al. 2021; Crippa et al. 2021; Guy and Flint 2004; Henson et al. 2021; Millar et al. 2018; Prieto González and Vila Silván 2021; Silmore et al. 2021; Stott et al. 2013; Torres-Moreno et al. 2018; Vitetta et al. 2021).

THC and CBD are mainly metabolized by CYP3A and CYP2C phase I enzymes in the liver and other tissues that express these enzymes, such as lung, intestine, and brain. THC is oxidized by these P450 (CYP450) isozymes to the psychoactive metabolite 11-hydroxy-THC (11-OH-THC) and then to the inactive metabolite 11-carboxy-THC (11-COOH-THC). CBD is oxidized to its active metabolite 7-OH-CBD, which is then oxidized to the inactive metabolite 7-COOH-CBD. These metabolites are excreted in feces and to a lesser extent urine after phase II metabolism by uridine 5′-diphospho-glucuronosyltransferase (UGT) enzymes (Fasinu et al. 2016, Millar et al. 2018, Huestis 2007, Lucas et al. 2018).

### Dosing guidelines

The recommended dosing of THC and/or CBD is to start at a low dose twice a day (or once daily for insomnia) and gradually titrate the dose over two to three weeks until the participant’s optimal dose is discovered. Reasons for this include to:

1. Minimize the occurrence of adverse effects that are dose dependent and for which tolerance often develops.
2. Allow attainment of steady state kinetics before the optimal dose is determined. Single-dose half-life is less than the steady state half-life that occurs when there is saturation of THC and CBD binding sites such as adipose tissue and blood proteins. Therefore, after approximately two weeks, less frequent dosing will be required for maintenance of effect than at the commencement of treatment.
3. Allow personalisation of optimal dose for effect and tolerability.

Dose may need to be adjusted as required for changes in indication severity, or changes in other medicines, foods or hepatic and renal function that may affect THC and CBD metabolism.

## Conclusion

THC and CBD combination medicines have a good safety and tolerability profile that is appropriate for opioid stage (stage 2–3) treatment of chronic pain. Low-dose CBD could be used as an initial treatment for chronic pain and for stress, anxiety, depression, and insomnia. High quality efficacy evidence is best for THC/CBD combination medicines for chronic pain and CBD-only medicines for stress and anxiety. The best mode of administration is an oral option that provides effective oral-buccal absorption and thus avoidance of first pass metabolism, otherwise the medicine is more 11-OH-THC and 7-OH-CBD than THC and/or CBD, and there is little or no data on the mechanism of action of the metabolites, 11-OH-THC and 7-OH-CBD.
